# Suitability of two distinct approaches for the high-throughput study of the post-embryonic effects of embryo-lethal mutations in Arabidopsis

**DOI:** 10.1038/s41598-017-17218-z

**Published:** 2017-12-05

**Authors:** Tamara Muñoz-Nortes, Héctor Candela, José Luis Micol

**Affiliations:** Instituto de Bioingeniería, Universidad Miguel Hernández, Campus de Elche, 03202 Elche, Spain

## Abstract

Several hundred genes are required for embryonic and gametophytic development in the model plant *Arabidopsis thaliana*, as inferred from the lethality of their mutations. Despite many of these genes are expressed throughout the plant life cycle, the corresponding mutants arrest at early stages, preventing the study of their post-embryonic functions by conventional methods. Clonal analysis represents an effective solution to this problem by uncovering the effects of embryo-lethal mutations in sectors of mutant cells within an otherwise normal adult plant. In this pilot study, we have evaluated the suitability of two sector induction methods for the large-scale study of the post-embryonic effects of embryo-lethal (*emb*) mutations in Arabidopsis. In line with the interests of our laboratory, we selected 24 *emb* mutations that damage genes that are expressed in wild-type vegetative leaves but whose effects on leaf development remain unknown. For the induction of mutant sectors in adult plants, we followed one approach based on the X-ray irradiation of ‘cell autonomy’ (CAUT) lines, and another based on the site-specific excision of transgenes mediated by Cre recombinase. We conclude that both methods are time-consuming and difficult to scale up, being better suited for the study of *emb* mutations on a case-by-case basis.

## Introduction

Mutational approaches have greatly advanced our understanding of developmental processes in plants and animals. The isolation and characterization of viable mutants with defective growth and pattern formation has been crucial to identify both housekeeping and regulatory genes that are required for the organism to attain its normal size and shape. By focusing on viable mutations, however, these screenings are likely to have missed many genes that play important post-embryonic roles, because they are essential in early developmental stages and there are not viable alleles to study. This is particularly important in plants, whose development takes place mostly post-embryonically, after the basic body plan is laid out during the embryogenesis. Post-embryonic development includes the development of important plant organs, such as the leaves. Indeed, numerous viable mutants identified in such screenings turned out to be hypomorphic (partial loss-of-function) alleles of genes otherwise known only by their embryonic lethal effects. Some examples are the *angulata1-1* (*anu1-1*), *anu7-1*, *anu9-1* and *scabra1-1* (*sca1-1*) mutants of *Arabidopsis thaliana* (hereafter, Arabidopsis), identified in a large-scale screen for viable mutants with abnormal leaf shape, size and pigmentation, which were later found to be hypomorphic alleles of the *SECA2*, *EMBRYO DEFECTIVE 2737* (*EMB2737*), *NON-INTRINSIC ABC PROTEIN 14* (*NAP14*) and *EMB3113* genes^1–3^. Another example is the *incurvata2-1* (*icu2-1*) mutant, identified in the same screen and found to be the first viable allele of the *ICU2* gene, which encodes the catalytic subunit of DNA polymerase α^4^. Because a significant fraction of the genes in the Arabidopsis genome is known to correspond to essential functions, and many such genes are expressed beyond the embryogenesis in wild-type plants, we hypothesized that many of them might also perform important roles in adult plants, after the embryogenesis has been completed.

Clonal analysis has been used to study embryo-lethal mutations by inducing genetic mosaics in many organisms, such as *Drosophila melanogaster*, maize and Arabidopsis^5–12^. Clonal analysis experiments typically combine a lethal gene and a cell-autonomous reporter gene or mutation with an easy-to-score phenotype, in order to identify induced mutant sectors that exhibit a post-embryonic mutant phenotype. By inducing mutant sectors in phenotypically wild-type plants, clonal analysis has helped researchers to answer questions regarding the phenotypic effects caused by the complete inactivation of embryo-lethal (*EMB*) genes in the tissues of an adult plant, the site of action of gene products, the cell autonomy and the cell lethality of lethal mutations. Several experimental approaches are available to perform clonal analysis in plants, including methods based on the loss of a chromosome arm after irradiation^6^, the mobilization of transposons^5^, or the use of transgenic approaches (e.g. based on the induction of site-specific recombinases to induce heritable changes in a given linage of cells^7,8,10,12,13^). In this work, we performed a pilot experiment aimed at determining which strategy is best suited for the high-throughput identification of the post-embryonic effects of a set of embryonic-lethal mutations. We tested two different strategies, one involving the use of X-rays and CAUT (cell autonomy) lines, and another based on the site-specific excision of transgenes mediated by Cre recombinase.

## Results and Discussion

In an attempt to select an efficient strategy that is suitable for the systematic identification of essential genes that also function post-embryonically, we have carried out pilot experiments using two different approaches aimed at inducing somatic sectors that express the mutant phenotype, one based on the use of CAUT lines^14^, and another based on the use of the Cre-*loxP* site-specific recombination system^7^. We focused on a subset of 24 *EMBRYO DEFECTIVE* (*EMB*) genes selected from the SeedGenes database (http://www.seedgenes.org/), which includes comprehensive information on the embryonic lethal genes of Arabidopsis^15^. *EMB* genes were selected based on the availability of embryo-lethal mutant alleles and on their expression patterns beyond the embryogenesis (Table 1), particularly focusing on genes that are expressed in wild-type leaves and basal rosettes (i.e. during the vegetative phase) according to publicly available data from the electronic Fluorescent Pictograph (eFP) browser database^16,17^. The genes selected encode proteins as diverse as transcription factors, proteasome subunits or epigenetic factors, which were considered good candidates to control leaf development at the transcriptional or post-transcriptional levels. We also selected some genes encoding proteins containing conserved domains whose functions remain unknown.

**Table 1:** *EMB* genes, CAUT lines and pCB1 constructs used in this work.

Gene name	AGI code	Chromosome	Coordinates	Protein function/conserved domains	Predicted location	Mutant allele	CAUT line	pCB1
*ATSWI3A*	AT2G47620	2	19531947–19534401	Subunit of SWI/SNF chromatin remodeling complex	Nucleus	*atswi3a-1*	7 F	Yes
*EMB1135*	AT1G79350	1	29844633–29853414	Orthologue of metazoan Strawberry notch (Sno) that mediates stress-induced chromatin memory	Nucleus	*emb1135*	C381	—
*EMB1381*	AT2G31340	2	13361506–13365200	Unknown function	Mitochondrion	*emb1381-1*	—	Yes
*EMB1408*	AT5G67570	5	26952352–26955543	Pentatricopeptide repeat-containing-protein involved in plastid gene expression	Chloroplast	*emb1408*	—	Yes
*EMB1441*	AT5G49930	5	20308033–20312808	Zinc knuckle (CCHC-type) family protein	Nucleus	*emb1441-1*	L82	Yes
*EMB1513*	AT2G37920	2	15868580–15870071	Copper ion transmembrane transporter	Plasma membrane	*emb1513-1*	—	Yes
*EMB1586*	AT1G12770	1	4351064–4353685	DEAD-box RNA helicase	Mitochondrion	*emb1586-1*	—	Yes
*EMB1611*	AT2G34780	2	14668653–14673904	Regulation of endoreduplication and maintenance of meristem cell fate	Plasma membrane	*emb1611*	L40	Yes
*EMB1637*	AT3G57870	3	21428496–21430200	SUMO ligase	Nucleus	*emb1637*	25_12	Yes
*EMB1674*	AT1G58210	1	21553621–21558056	Member of the NET superfamily that couples membranes to the actin cytoskeleton	Plasma membrane	*emb1674-1*	—	Yes
*EMB1688*	AT1G67440	1	25263804–25265719	Minichromosome maintenance (MCM) family protein	Chloroplast	*emb1688-1*	—	Yes
*EMB1691*	AT4G09980	4	6247735–6252288	Required for N6-adenosine methylation of mRNA	Nucleus, cytoplasm	*emb1691-1*	L104	Yes
*EMB1706*	AT4G10760	4	6619817–6623351	Required for N6-adenosine methylation of mRNA	Nucleus	*emb1706-1*	L104	Yes
*EMB1745*	AT1G13120	1	4469181–4473213	Nucleoporin GLE1-like protein	Nuclear envelope	*emb1745*	—	Yes
*EMB1895*	AT4G20060	4	10854790–10859330	Armadillo (ARM)-repeat superfamily protein involved in small nuclear RNAs (snRNA) 3′ end maturation	Nucleus	*emb1895-1*	—	Yes
*EMB1923*	AT4G28210	4	13990617–13992078	Unknown function	Chloroplast	*emb1923-1*	L4	—
*EMB1990*	AT3G07430	3	2379193–2380198	YGGT family protein involved in nucleoid distribution	Chloroplast	*emb1990-1*	C413	Yes
*EMB2001*	AT2G22870	2	9739457–9741104	P-loop containing nucleoside triphosphate hydrolases superfamily protein	Cytoplasm	*emb2001-1*	30B4	Yes
*EMB2036*	AT5G66055	5	26417156–26419264	Ankyrin repeat protein	Chloroplast	*emb2036-1*	—	Yes
*EMB2107*	AT5G09900	5	3089278–3092595	Isoform of the 26 S proteasome regulatory protein subunit RPN5	Nucleus, cytoplasm	*emb2107*	—	Yes
*EMB2301*	AT2G46770	2	19220727–19222916	Transcription factor	Nucleus	*emb2301*	7 F	—
*EMB2410*	AT2G25660	2	10916203–10927390	Unknown function	Chloroplast	*emb2410-1*	30B4	—
*EMB2736*	AT3G19980	3	6961736–6965108	Catalytic subunit of serine/threonine protein phosphatase 2A	Nucleus, plasma membrane, cytoplasm	*emb2736*	—	Yes
*EMB3008*	AT5G39750	5	15906875–15907942	MADS-box transcription factor	Nucleus	*emb3008*	B111	Yes

### Sector induction using CAUT lines and X-rays

**Figure 1:**
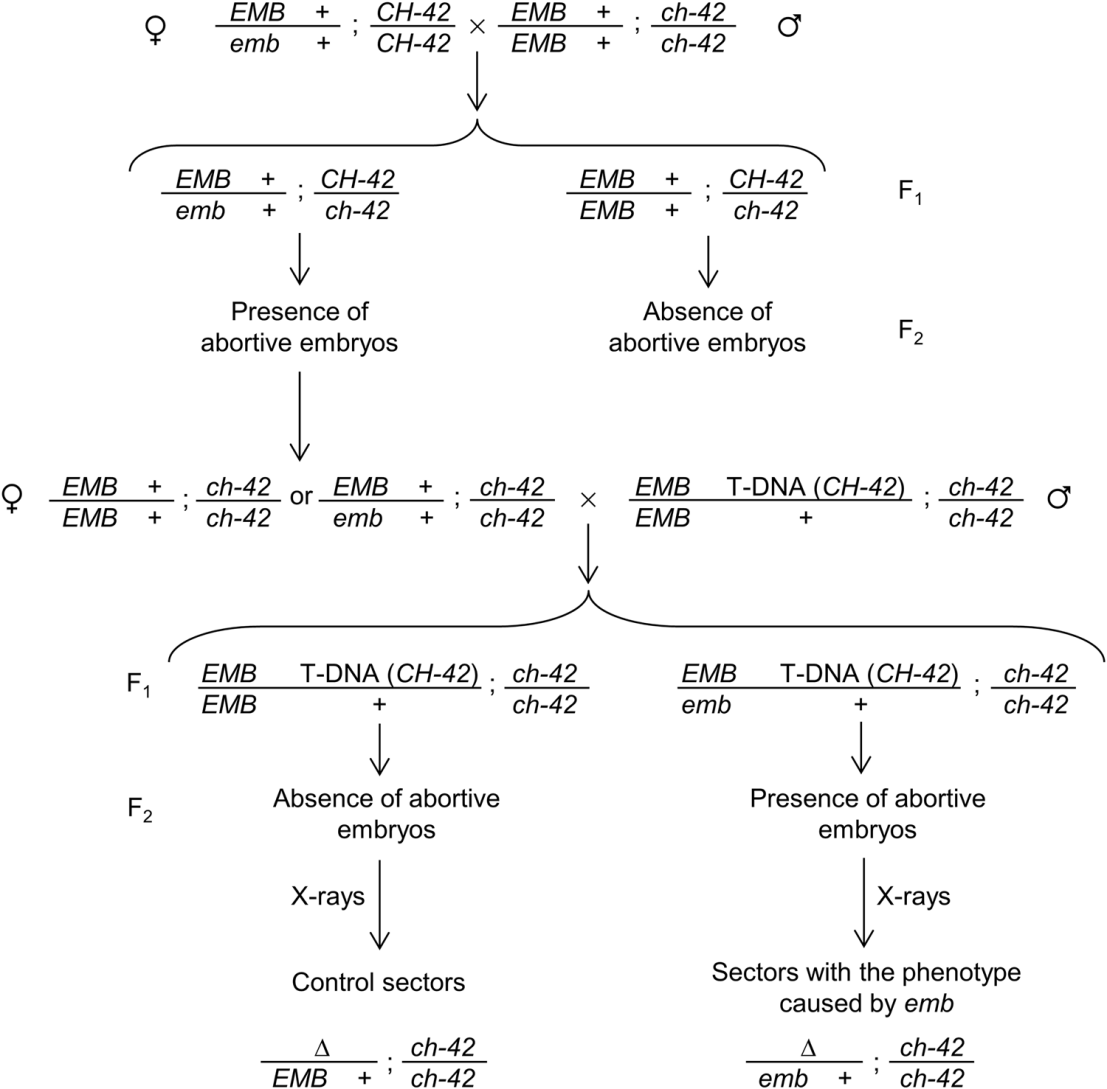
Detailed strategy to obtain hemizygous sectors for an embryo-lethal (*emb*) mutation by means of X-rays. Only the relevant genotype of each member from a pair of homolog chromosomes is indicated. The generation derived from a cross is indicated as F_1_, and the progeny of its self-fertilization is indicated as F_2_. The uppercase Greek letter delta (∆) represents the loss of a chromosome fragment. In cells with the appropriate genotype, the loss of a chromosome fragment containing the *CHLORATA-42* (*CH-42*) transgene and the wild-type copy of the *EMB* gene gives rise to a cell with pale-green genotype which might be accompanied by a mutant phenotype caused by the *emb* mutation.

**Figure 2:**
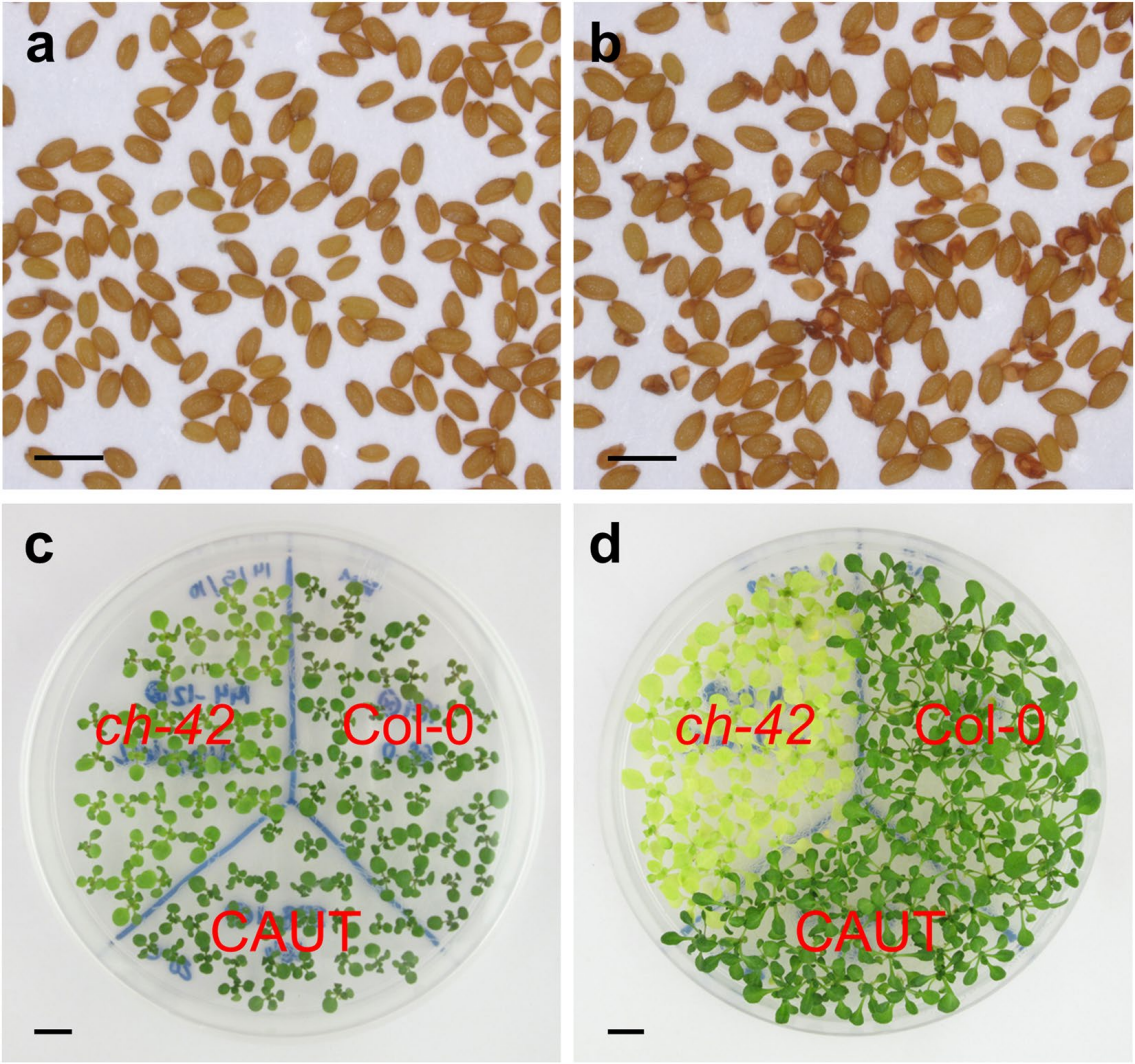
Selection of *EMB*/*emb* lines and effects of temperature on *ch-42* plants. (**a**,**b**) F_2_ mature seeds derived from a cross involving *EMB*/*emb*;*CH-42*/*CH-42* and *EMB*/*EMB*;*ch-42*/*ch-42* plants. (**a**) Absence of abortive seeds indicates that the F_2_ line does not carry the *emb* mutation, and (**b**) presence of abortive seeds indicates that the F_2_ line carries the *emb* mutation. (**c**,**d**) Plants from different genotypes growing at (**c**) 20 °C, and (**d**) 26 °C. Scale bars represent (**a**,**b**) 1 mm, and (**c**,**d**) 1 cm.

For the induction of marked somatic sectors in Arabidopsis, we initially took advantage of the availability of CAUT lines with insertions located on every chromosome arm^14^. Thirteen different *EMB* genes (Table 1) were selected based on the availability of suitable CAUT lines carrying an insertion of the *CHLORATA-42* (*CH-42*) gene located between the *EMB* gene and the centromere of the corresponding chromosome. *CH-42* encodes the CHLI subunit of magnesium chelatase, which is required for chlorophyll biosynthesis. By choosing this configuration, we expect that all marked (yellow) sectors found after X-ray irradiation have also lost the wild-type allele of the *EMB* gene. To implement this strategy (Fig. 1), we systematically crossed heterozygous *EMB*/*emb* plants to the homozygous *ch-42*/*ch-42* mutant and isolated F_2_ plants displaying the recessive yellow phenotype caused by *ch-42*. The presence of aborted embryos or collapsed seeds in the siliques of these plants allowed us to select *ch-42*/*ch-42* plants segregating the corresponding *emb* mutation in the F_3_ progeny (Fig. 2a,b). Plants with the *EMB*/*emb*; *ch-42/ch-42* genotype were subsequently crossed to appropriate CAUT lines. Ten different CAUT lines were used for this purpose (Table 1). Whenever possible, we selected CAUT lines carrying the *CH-42* insertion that maps closest to the *EMB* gene, because a higher frequency of chromosomal breaks is expected to occur as the distance between the insertion and the centromere increases. This crossing scheme allowed us to select phenotypically wild-type (green) plants that carry an insertion of the *CH-42* transgene in the F_2_ generation. F_3_ families segregating individual *emb* mutations were then established from F_2_ plants that had aborted embryos in their siliques. Sibling families not segregating the *emb* mutations were also established from each cross as a control. We tested the Mendelian segregation of the yellow *ch-42* phenotype in these F_3_ families. Unexpectedly, we found a high number of plants exhibiting a yellow phenotype in seven (out of the thirteen) families segregating aborted seeds, suggesting that the *CH-42* transgene fails to complement the *ch-42* allele (possibly due to silencing) or that it is located at a higher-than-expected chromosomal distance from the corresponding *EMB* gene.

In phenotypically wild-type *ch-42*/*ch-42*; *EMB CH-42*/*emb* – plants, X-rays can cause chromosomal breaks between the centromere and the T-DNA insertion, and are expected to generate hemizygous yellow sectors when the acentric fragment carrying the extra copy of *CH-42* and the *EMB* wild-type allele is lost. A drawback of irradiating F_3_ families, which comprise seeds with a mixture of genotypes, is that recombination events between the loci of the T-DNA insertions and the linked *EMB* genes might lead to yellow sectors that still keep a functional copy of the *EMB* gene. Any developmental or other visible phenotypes occurring specifically in the yellow sectors can be attributed to the post-embryonic effects of the corresponding *emb* mutation only if they are not observed in the irradiated control families. Because the cells in the L1 layer are colorless and those in the L3 contribute comparatively little to most organs, the *ch-42* yellow phenotype is best scored in the cells of the L2 layer, making this marker most useful for the study of genes that function in this layer^14^.

**Figure 3:**
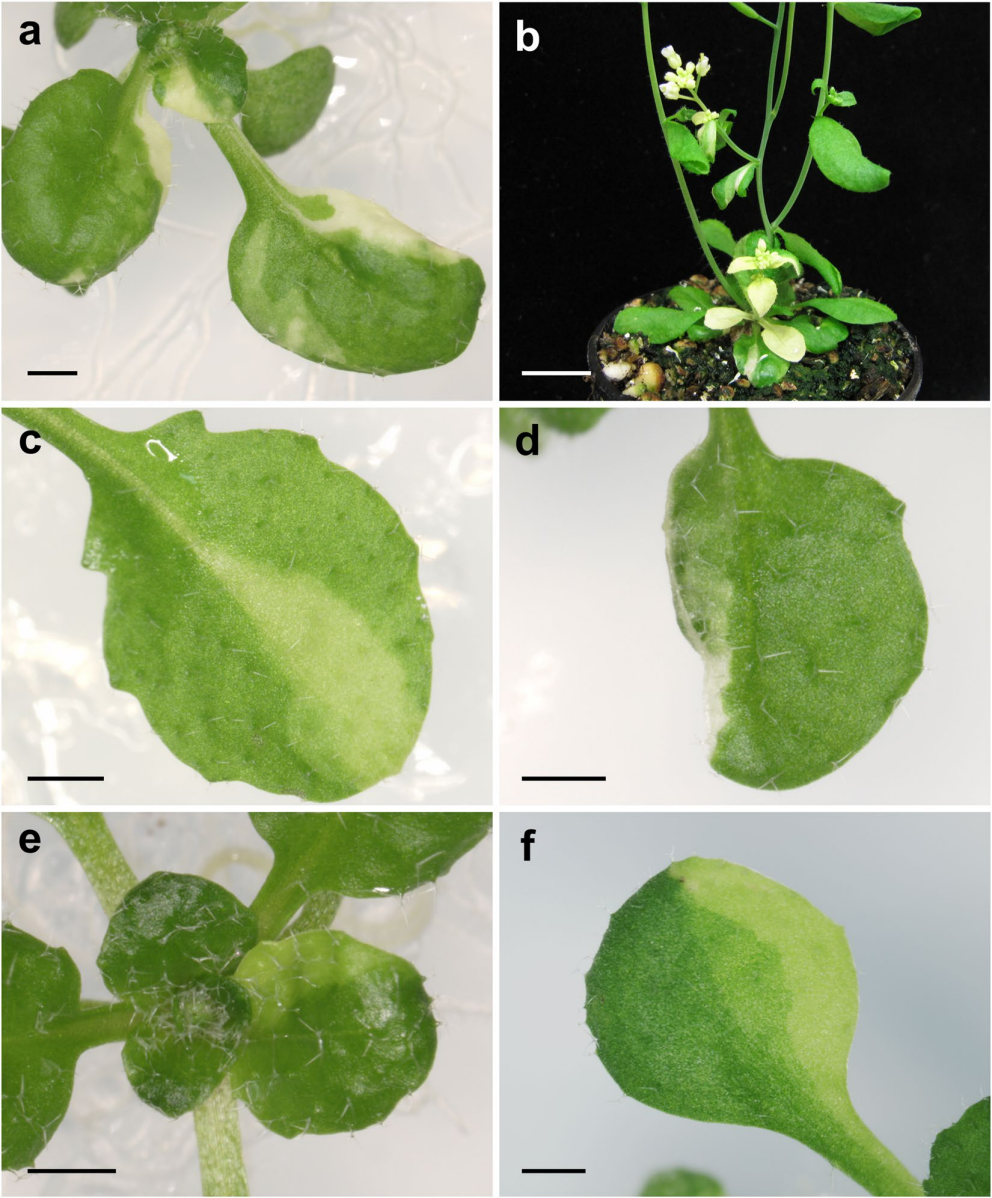
Sectors identified after X-rays irradiation. Plants from irradiated families segregating (**a**,**b**) *emb1441*, (**d**) *emb2001*, and (**e**) *emb1706* mutations. (**c**,**f**) Plants from irradiated families that are not segregating *emb* mutations. Plants were irradiated at dosages of (**a**–**c**) 1000 and (**d**–**f**) 16000 rad. Plants were collected (**a**,**c**–**f**) 14 and (**b**) 40 days after stratification. Scale bars represent (**a**,**c**–**f**) 1 mm and (**b**) 1 cm.

Two different X-ray dosages were used to induce sectors. On the one hand, water-imbibed seeds were subjected to a dosage of 1000 rad (10 Gy) based on previous reports from the Arabidopsis and maize literature^6^. On the other, dry seeds received a dosage of 16000 rad (160 Gy), as previously described^14^. The irradiation of dry seeds allowed us to stagger the sowing of the irradiated families. Plants were periodically examined under the stereomicroscope to identify yellow sectors. The temperature sensitivity of the *ch-42* mutation, which determined a paler pigmentation at 26 °C than at 20 °C, made the yellow sectors easier to spot and helped us to select plants with the correct genotype (Fig. 2c,d). Sectors occurred at a very low frequency in the families irradiated at 1000 rad (Fig. 3a–c). In these families, we only found 6 sectors, one half of which appeared in control families lacking an *emb* mutation (Fig. 3c). Three of these sectors, including two in the control families, were completely albino, rather than yellow, suggesting that rearrangements caused by X-rays lead to visible phenotypes even when *emb* mutations are not involved. By contrast, we found sectors in every family in about 1% of the plants irradiated at 16000 rad (Fig. 3d–f), a frequency that is roughly similar to the frequency reported by Furner *et al*.^14^. In the six families that exhibited a clear distortion of the Mendelian segregation of the yellow phenotype caused by *ch-42*, we found somatic sectors in both types of irradiated families (segregating and not segregating the *emb* mutation; Fig. 3f), making it difficult to draw conclusions on the post-embryonic roles of the corresponding genes.

**Figure 4:**
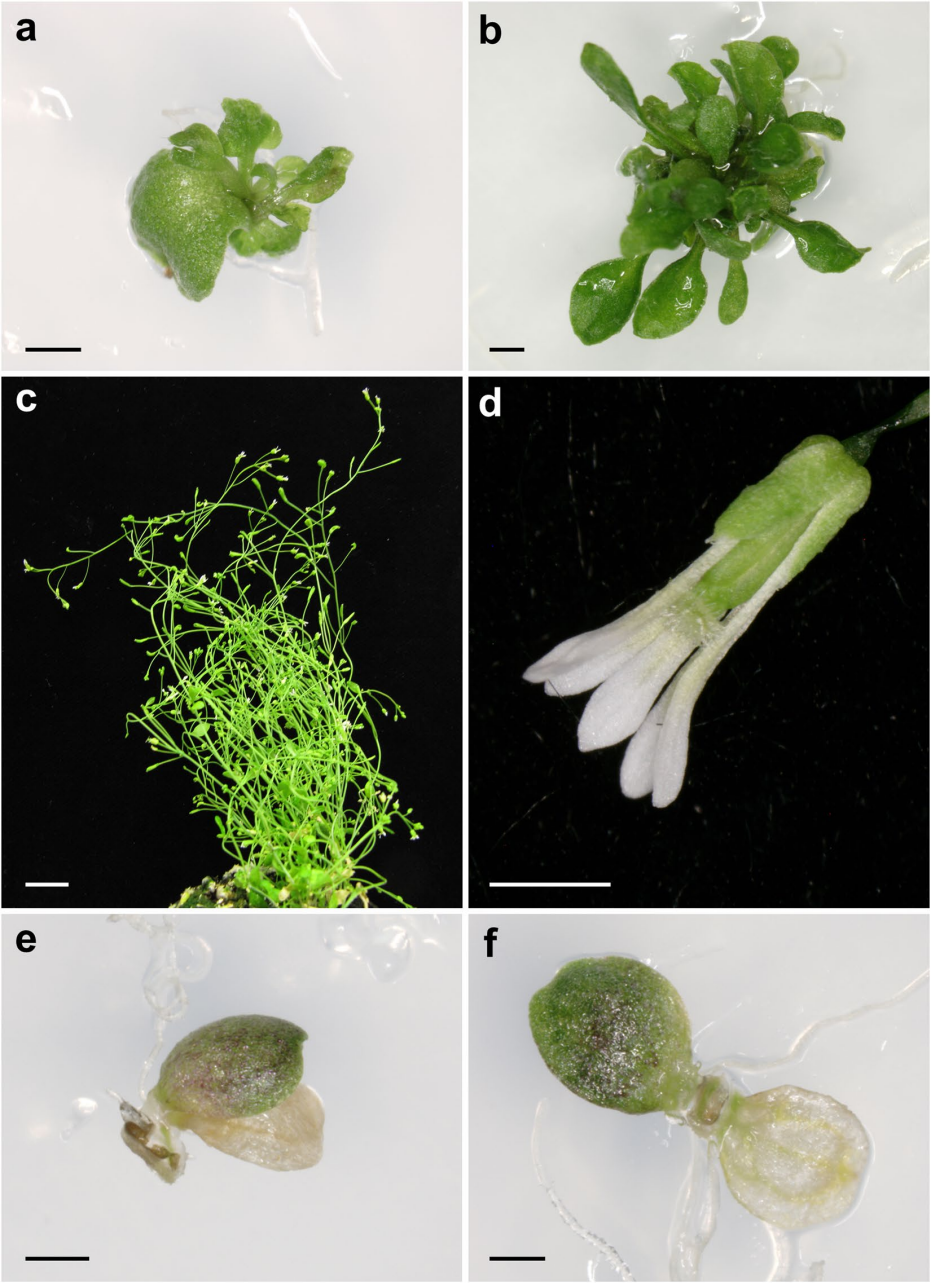
Putative escapers for (**a**) *emb1135*, (**b**–**d**) *emb1706*, and (**e**,**f**) *emb2410* mutations. Plants were collected (**a**,**e**,**f**) 21, (**b**) 40 and (**c**,**d**) 50 days after stratification. Scale bars represent (**a**,**b**,**d**–**f**) 1 mm, and (**c**) 1 cm.

Incidentally, this approach occasionally allowed us to find escapers for some *emb* mutations, i.e. plants that completed the embryogenesis and reached the seedling stage or beyond, potentially providing information on the post-embryonic function of the genes. Escapers were found for mutant alleles of three *EMB* genes (Fig. 4), in all cases at a very low frequency in the F_2_ generation (0.72% for *emb1135*, 1.92% for *emb1706-1*, and 0.48% for *emb2410-1*). The majority of escapers were pale green, as expected from our crossing scheme, and exhibited additional developmental phenotypes. Although we did not genotype the T-DNA insertions in the escapers, the observed phenotypes were absent from the control families (which lacked collapsed seeds), suggesting that they were specifically caused by the loss of a given *EMB* gene. The *emb1135* escapers were small, with fused cotyledons, wrinkled surface and irregular margins (Fig. 4a). The *emb2410* escapers expanded their cotyledons and then died (Fig. 4e,f). The *emb1706* escapers formed small rosettes, which included leaves with long petioles and adaxially curved leaf laminae (Fig. 4b). When transferred to soil, the *emb1706* escapers produced numerous secondary shoots (Fig. 4c) with abnormally patterned flowers (Fig. 4d).

### Sector induction using Cre recombinase

**Figure 5:**
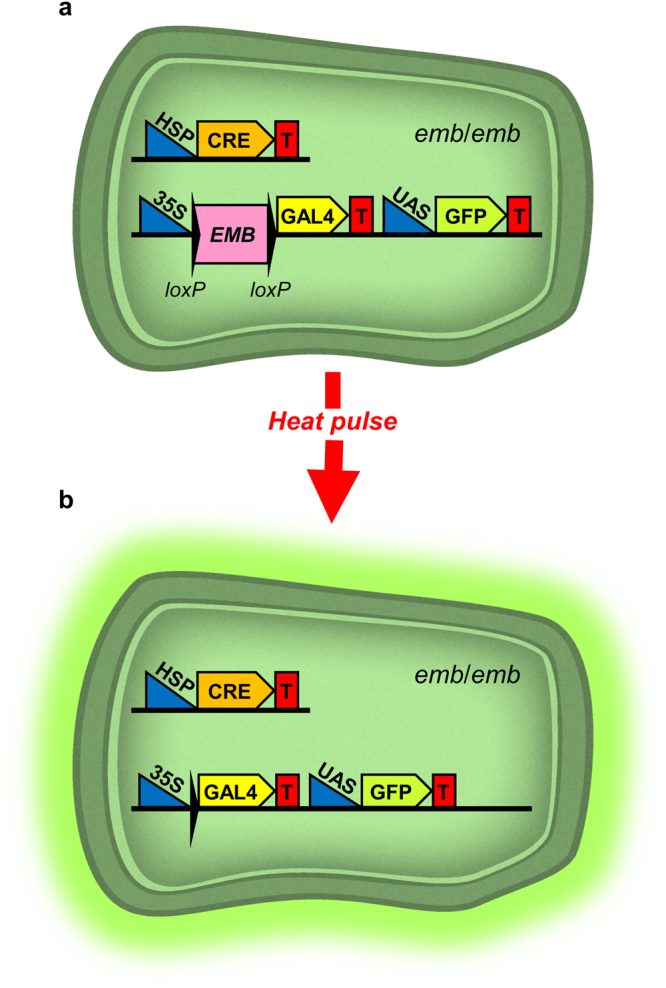
Transgene-mediated approach to generate hemizygous marked sectors for embryo-lethal mutations. (**a**) Cell with the appropriate genotype for induction of fluorescent sectors by heat shock. This cell is homozygous for the embryo-lethal mutation (*emb*/*emb*) and carries two different constructs, one of them providing a wild-type copy of an *EMB* gene that allows its normal development, and the other with a heat-shock promoter driving the inducible expression of Cre recombinase. (**b**) A heat pulse causes the activation of Cre and a concomitant loss of the wild-type copy of the *EMB* gene through the excision of the Gateway cassette mediated by the action of Cre recombinase on the *loxP* sites. The subsequent action of GAL4 on the UAS drives the expression of GFP and marks the cell, which is fluorescent and might exhibit any mutant phenotype associated with the loss of function of the *EMB* gene in adult tissues.

**Figure 6:**
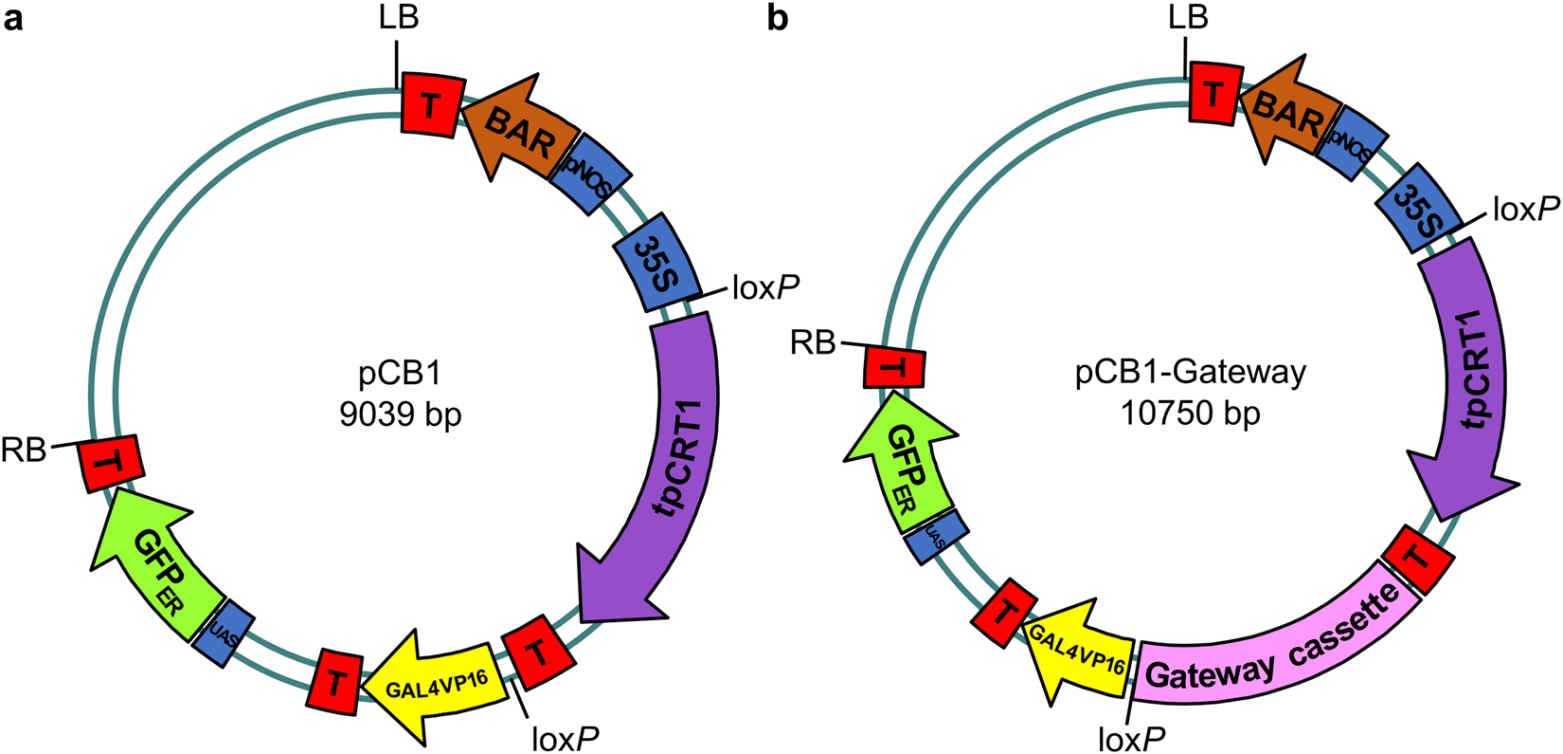
Maps of pCB1 and pCB1-Gateway vectors. (**a**) The pCB1 binary vector, and (**b**) the modified pCB1-Gateway vector. LB: T-DNA left border; T: transcriptional terminator; BAR: bialaphos resistance gene; pNOS: nopaline synthase promoter; 35 S: constitutive promoter; lox*P*: Cre recombination site; tpCRT1: resistance gene; GAL4VP16: transcriptional activator; UAS: upstream activating sequence; GFP_ER_: endoplasmic reticulum-localized green fluorescent protein; RB: T-DNA right border.

We also tested a strategy based on the site-specific excision of transgenes driven by a heat-inducible Cre recombinase (Fig. 5). To this end, we prepared two Gateway-compatible versions of the pCB1 vector (see Material and Methods), which is intended for the induction of clonal sectors by means of the Cre-mediated excision of a cassette containing a wild-type copy of the gene of interest (Fig. 6). We used the Gateway cloning technology to systematically create 20 entry clones, each containing a different genomic region able to complement the embryonic lethality of a selected *emb* mutation (Table 1). These entry clones were transferred to the Gateway-compatible version of pCB1 using LR reactions in order to obtain constructs for plant transformation. Because the Gateway cassette is flanked by two *loxP* sites, the genomic inserts of these constructs can be excised by expressing Cre to produce GFP-marked, *emb*/*emb* mutant sectors.

**Figure 7:**
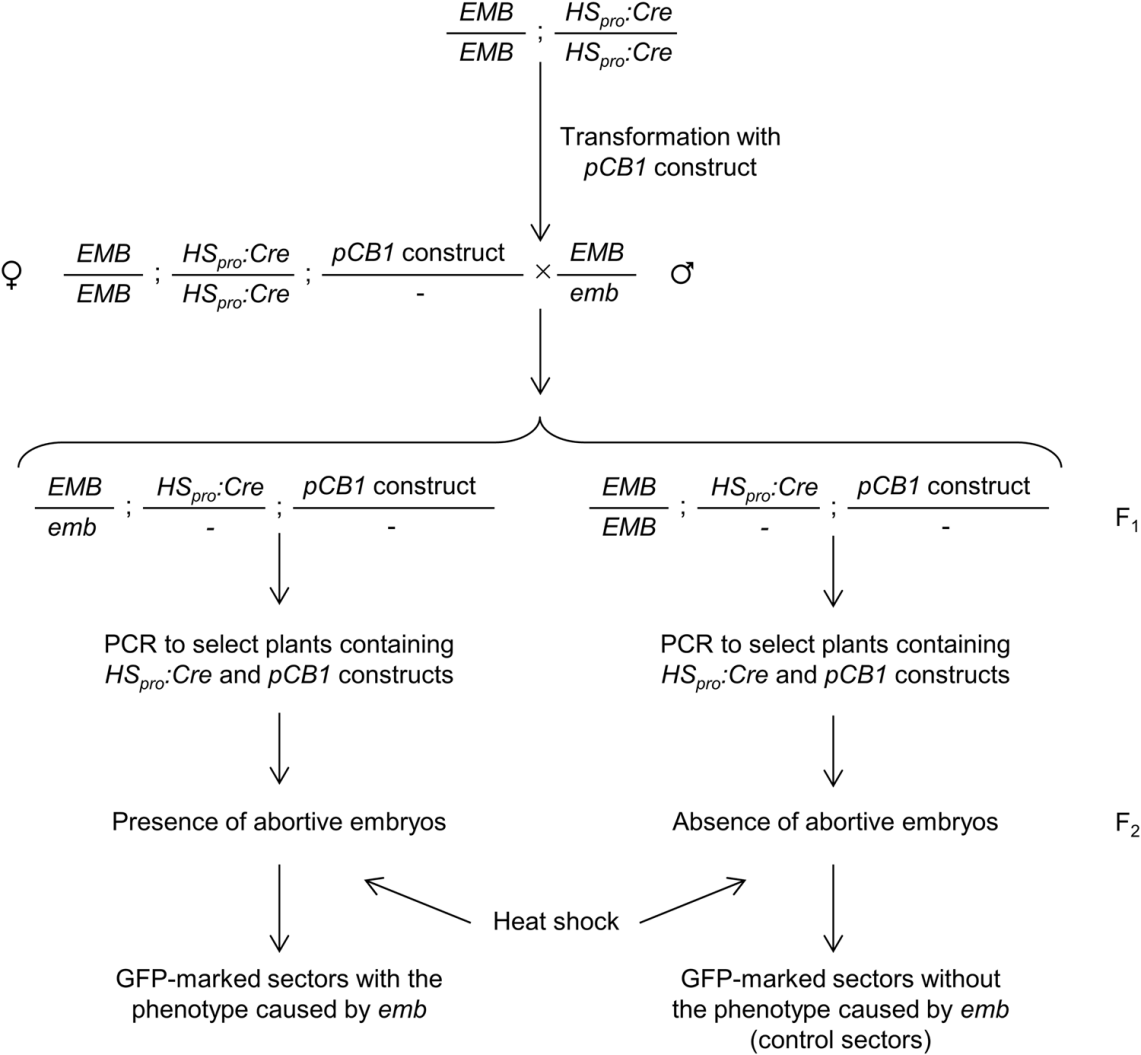
Detailed strategy to obtain GFP-marked sectors which are hemizygous for an embryo-lethal (*emb*) mutation by means of a heat-shock. Only the relevant genotype of each member from a pair of homolog chromosomes is indicated. The generation derived from a cross is indicated as F_1_, and the progeny of its self-fertilization is indicated as F_2_. In cells with the appropriate genotype, the activation of Cre recombinase causes the excision of the wild-type copy of the *EMB* gene and gives rise to a cell marked with GFP that exhibits an additional mutant phenotype caused by the *emb* mutation.

**Figure 8:**
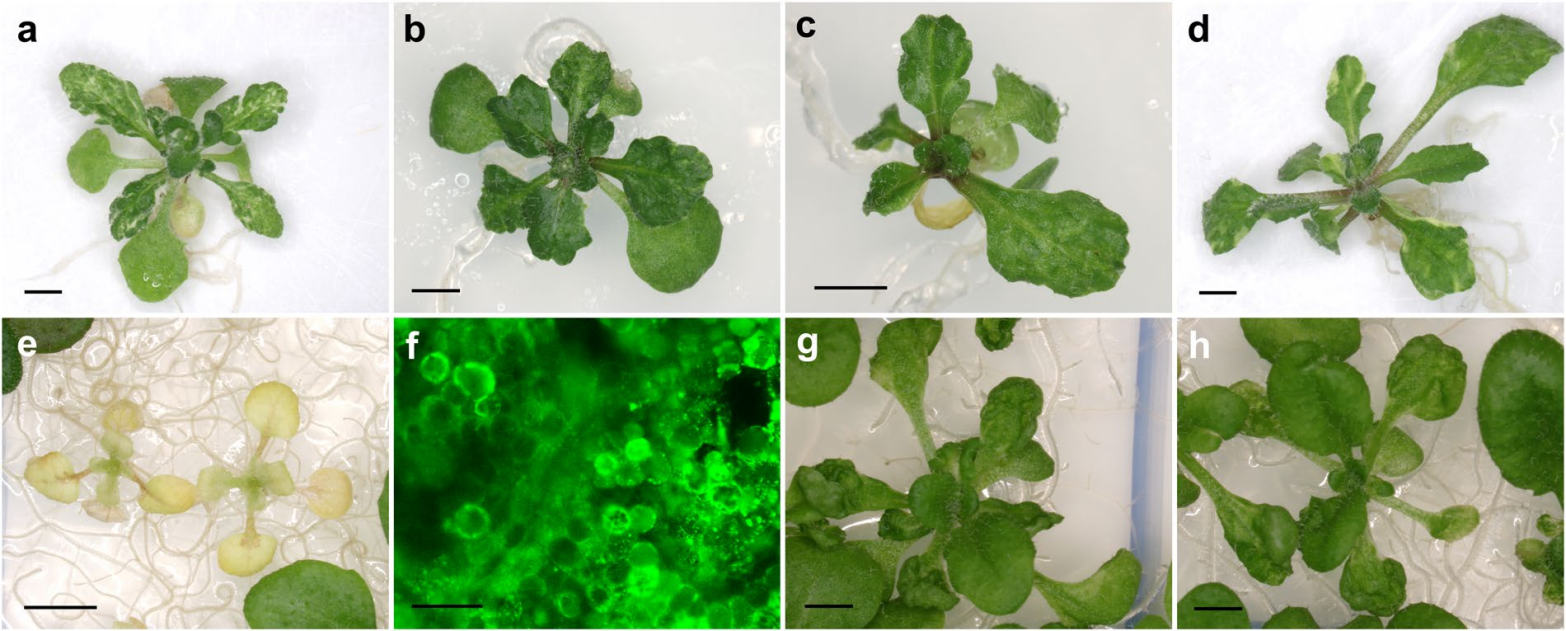
Observed phenotypes after inducing sectors by heat shock. (**a**–**f**) Plants carrying (**a**) *emb1408*, (**b**) *emb1586*, (**c**) *emb1637* and (**d**,**e**) *emb2001* mutations. (**g**,**h**) Control plants carrying the *HS*_pro_:*Cre* and pCB1-Gateway constructs, but not an *emb* mutation. (**e**) Pale-green plants with impaired growth. (**f**) Intense GFP fluorescence in one of the plants shown in (**e**). Scale bars represent (**a**–**e**, **g**,**h**) 2 mm, and (**f**) 50 µm.

In order to obtain transgenic lines for 20 non-allelic *emb* mutations (Fig. 7), we first transformed homozygous *HS*_*pro*_ *:Cre* plants with the pCB1-Gateway constructs, each carrying a wild-type copy of a different *EMB* gene. The resulting T_1_ transformants are expected to carry insertions of two T-DNAs, one from the pCB1-Gateway vector and another to allow the inducible expression of Cre driven by a heat shock promoter. These transgenic plants were subsequently crossed to *EMB*/*emb* heterozygotes to isolate plants carrying the *emb* mutation and both constructs. The F_1_ and F_2_ progenies of these crosses were genotyped by PCR to verify the presence of both constructs before sector induction. Ideally, the induction of informative sectors should be performed on plants homozygous for the *emb* mutation and hemizygous for the pCB1-Gateway construct, which would require additional generations and a complex crossing scheme before the plants can be heat-shocked. For this reason, we induced the sectors directly in the F_2_ plants, some of which must have the desired genotype, although at the expense of screening a larger plant population. Plates containing 6-days-after-sowing F_2_ seedlings were sealed with Parafilm and heat-shocked for 30 min at 37 °C in a water bath. We reproducibly found leaf sectors for four different *emb* mutations: *emb1408-1*, *emb1586-1*, *emb1637-1* and *emb2001-1* (Fig. 8). However, sectors similar to those for *emb1637-1* and *emb2001-1* occurred in the corresponding control lines (i.e. lines carrying the pCB1-Gateway and *HS*_*pro*_ *:Cre* constructs but lacking an *emb* mutation, which we selected in parallel based on the absence of segregating collapsed seeds; Fig. 8g,h), showing that sectors with a mutant phenotype can arise from Cre-induced chromosomal rearrangements even in the absence of an embryonic-lethal mutation. In addition to these, we found some heat-shocked families segregating plants with impaired growth and a chlorotic phenotype (Fig. 8e). These plants exhibited intense and generalized GFP fluorescence (Fig. 8f), questioning whether the observed phenotypes were indeed caused by the loss of a specific *EMB* gene or if they were instead due to deleterious, non-specific consequences of elevated Cre expression in the affected tissues.

## Concluding Remarks

In this report, we have tested two different strategies for the induction of somatic sectors in adult plants. The first approach, based on the use of CAUT lines, did not scale up well for high-throughput studies. In addition to being labour-intensive and time-consuming, this strategy required a complex crossing scheme with several generations before plant materials were ready for irradiation. According to Furner *et al*.^14^, the timing required for preparing a single line is about 40 weeks. This approach is further complicated when the *emb* mutations reside in the same chromosome arm as the *ch-42* marker (on chromosome 4) or when they map very close to a centromere. The latter problem might make it difficult to identify an appropriate CAUT line for a given *emb* mutation, and a short distance between the *CH-42* transgene and the centromere is expected to result in a low frequency of sectors. Furthermore, scoring the boundaries of yellow sectors is a problematic task, particularly when the sectors are small or hard to distinguish from other pale-green necrotic sectors that occur non-specifically (i.e. which might also be present in control families) as a secondary effect of the X-ray treatment.

Implementing the second strategy, based on the use of the site-specific Cre recombinase and transgenes, was more straightforward. To establish an efficient cloning pipeline, we first prepared a Gateway destination vector based on the pCB1 vector, which has previously been used effectively to characterize the effects of individual embryonic-lethal mutations in somatic sectors in *Arabidopsis thaliana* ^7^. We found that a skilled operator can efficiently streamline the making of entry clones containing large genomic inserts by using high-fidelity DNA polymerases (e.g. Phusion High-Fidelity DNA Polymerase) and primers containing *att*B1 and *att*B2 sites for subsequent recombination into the Gateway-compatible version of pCB1. However, scaling up this approach was also time-consuming because, similar to the approach based on CAUT lines, it required crossing, genotyping and propagating the plants for several generations before obtaining families with an adequate genotype for sector induction. Fine-tuning the X-ray dosages or the duration of the heat-shock treatment should help to minimize the secondary effects of both treatments while optimizing the frequency of somatic sectors specifically being due to loss of *EMB* functions.

Additional information might be obtained from the characterization of hypomorphic (non-null) alleles of *EMB* genes, which might be difficult to isolate, or from the complementation of null alleles with transgenes carrying a copy of the corresponding wild-type *EMB* gene driven by an embryo-specific promoter, two approaches that have been successfully applied to the study of individual genes. As an example, weak mutations in *EMB2107* and *EMB1611* ^18,19^ have recently been found to cause post-embryonic phenotypes in leaves. Such alleles would be ideal controls in future clonal analysis experiments with a larger number of plants or aimed at defining an optimal set of experimental conditions.

## Methods

### Plant materials, growth conditions and crosses

Seeds of the *Arabidopsis thaliana* L. Heynh. wild-type accessions Landsberg *erecta* (L*er*) and Columbia-0 (Col-0), as well as heterozygous *EMB*/*emb* lines and CAUT lines (Table 1) were obtained from the Nottingham Arabidopsis Stock Centre (NASC; http://arabidopsis.info/). Transgenic seeds carrying the *HS*_*pro*_ *:CRE* construct were kindly supplied by Dr. Guy Wachsmann. Seed sterilization, sowing, plant culture and crosses were performed as previously described^20,21^. Briefly, seeds were sown on plates containing Murashige and Skoog (MS) agar medium [half-strength MS salts, 0.7% plant agar (Duchefa), pH 5.7, and 1% sucrose], stratified at 4 °C in the dark for 24 h and then transferred to TC16 or TC30 growth chambers (Conviron) set to our standard conditions (continuous light at approximately 75 μmol·m^−2^·s^−1^, 20 °C, 60-70% relative humidity). When required, plants were transferred to pots containing a 2:2:1 mixture of perlite:vermiculite:sphagnum moss and grown in walk-in growth chambers set to our standard conditions. For selection of transgenic plants, T_1_ seeds were sown in flat pots containing perlite and river sand and were sub-irrigated with ATM supplemented with 15 mg/l glufosinate ammonium (Finale).

### Irradiation and sector screening

Irradiation of Arabidopsis seeds was performed using a Philips MG102 X-ray cabin. Seeds were irradiated at doses of 10 Gy for sterilized seeds and 160 Gy for dry seeds, as previously described^6,14^. At least two control wild-type lines and two heterozygous *EMB*/*emb* lines of each of the 13 genotypes were irradiated. After irradiation, seeds were sown in Petri dishes and the resulting plants were checked periodically, looking for mutant sectors. Pictures of the different sectors were taken, and the leaves that contained them were collected and stored. Plants containing sectors were moved to soil pots in order to verify if they spread to other plant organs like secondary shoots, cauline leaves or flowers, as previously described^14^.

### Modification of pCB1 vector

We modified the pCB1 vector^7^ for use with the Gateway cloning technology (Fig. 6). For this, pCB1 was linearized with *Not*I, and the resulting cohesive ends were filled in with Klenow to generate blunt ends. A PCR product corresponding to a Gateway cassette (Frame A) was amplified with Phusion DNA polymerase (Finnzymes) and ligated to pCB1 using T4 ligase (Fermentas). The ligation products were transformed into the *Escherichia coli* DB3.1 strain, and colonies resistant to both kanamycin and chloramphenicol were selected. This modified plasmid was called pCB1-Gateway. After purifying the plasmids that carried the insert of interest, its orientation was checked with a *Sma*I and *Sal*I double digestion. We obtained two different versions of the pCB1-Gateway vector, with the Gateway cassette oriented in both possible directions, (+) and (−).

### Generation of pCB1-Gateway constructs

In order to introduce a wild-type copy of the *EMB* genes of interest into the pCB1-Gateway empty vector, we amplified genomic regions containing each *EMB* gene spanning from the end of the previous gene coding region to the beginning of the following gene coding region, to make sure that the regulatory sequences were also included. We designed primer pairs containing *att*B1 and *att*B2 sites (Table 2), in order to amplify the regions that contain each *EMB* gene of interest from its corresponding bacterial clone. These regions were PCR amplified using the Phusion polymerase (Finnzymes). The amplification products were purified and used in different BP reactions (Invitrogen), in which the pGEM-T Easy 221 plasmid was used as entry vector. Chemocompetent DH5*α Escherichia coli* cells were transformed by heat shock with the products of BP reactions. Colonies carrying the pGEM-T Easy 221 plasmid were selected in Petri dishes with LB medium supplemented with ampicillin. Insert presence was checked by rapid size screen with lysis buffer^22^, digestion with the restriction enzyme *Not*I and PCR with plasmid and insert primers (Table 2). Positive colonies were used to perform LR reactions (Invitrogen) with the appropriate pCB1-Gateway destination vector. Each LR reaction was performed twice, using the pCB1-Gateway plasmids with the Gateway cassette in both orientations. Chemocompetent DH5*α Escherichia coli* cells were transformed by heat shock with the LR products and colonies carrying the pCB1-Gateway vector were selected in LB medium supplemented with kanamycin. The presence of each insert was checked by double digestion with *Xba*I and *Sma*I restriction enzymes. Positive clones were mobilized into *Agrobacterium tumefaciens* C58C1 pSOUP cells by electroporation. Every pCB1-Gateway construct was transferred to plants carrying the *HS*_*pro*_ *:CRE* construct by the floral dip method^23^.

**Table 2:** Primers used in this work.

Gene name	Amplified region (bp)	Primers
*ATSWI3A*	4001	F: ggggacaagtttgtacaaaaaagcaggctACTTTCAGGTTGTTCACCAGA
		R: ggggaccactttgtacaagaaagctgggtTCTCACGTATTCCTGTCACCA
*EMB1381*	5694	F: ggggacaagtttgtacaaaaaagcaggctTTGGACCGTAATAACATCCCG
		R: ggggaccactttgtacaagaaagctgggtCAAAAGAGAATCCATTTCCAC
*EMB1408*	5191	F: ggggacaagtttgtacaaaaaagcaggctCGATCAAGCTTTGGGATCTCG
		R: ggggaccactttgtacaagaaagctgggtCCGAATATGAAAAGGCATGTC
*EMB1441*	8456	F: ggggacaagtttgtacaaaaaagcaggctGCTCAATTGGTAGTTGTTCTG
		R: ggggaccactttgtacaagaaagctgggtTACAAGGCCCACCCAAAGTTT
*EMB1513*	4593	F: ggggacaagtttgtacaaaaaagcaggctAGGCGTAAGCTCACTGTGTTG
		R: ggggaccactttgtacaagaaagctgggtTTCGAAAGAAAAATCCGACAA
*EMB1586*	3901	F: ggggacaagtttgtacaaaaaagcaggctGTGTTCATGACCCACGACATT
		R: ggggaccactttgtacaagaaagctgggtTTTGGCAATGGCACTAAACAA
*EMB1611*	7464	F: ggggacaagtttgtacaaaaaagcaggctCCTGGAAACATGACTTCGGTC
		R: ggggaccactttgtacaagaaagctgggtGGCCAGTAAAACCACCAAACC
*EMB1637*	4001	F: ggggacaagtttgtacaaaaaagcaggctGGTGGTGGTTTGTTGCCTTCT
		R: ggggaccactttgtacaagaaagctgggtGGGTTGGTTGCTGTTGAGATT
*EMB1674*	6435	F: ggggacaagtttgtacaaaaaagcaggctCACGCATGCAACAGAGATGAC
		R: ggggaccactttgtacaagaaagctgggtATGGCTCCTCTCTCCAAAGGA
*EMB1688*	3384	F: ggggacaagtttgtacaaaaaagcaggctGTGACTTGTTGTTTTGGTTAG
		R: ggggaccactttgtacaagaaagctgggtTTGAACTATCACGTCTTTTCC
*EMB1691*	7693	F: ggggacaagtttgtacaaaaaagcaggctGCCGGGTAGAGAAATACACTG
		R: ggggaccactttgtacaagaaagctgggtACCAATTTGTGGTGCGGTTGC
*EMB1706*	8220	F: ggggacaagtttgtacaaaaaagcaggctATCTCCTTCAAAGTTCAGCTC
		R: ggggaccactttgtacaagaaagctgggtATCTTGCTTGTGAGAAAGGCA
*EMB1745*	6539	F: ggggacaagtttgtacaaaaaagcaggctTGCAGGAGTAAACACAAGCGC
		R: ggggaccactttgtacaagaaagctgggtATAGAGAGAGGGTTGAGGAG
*EMB1895*	8109	F: ggggacaagtttgtacaaaaaagcaggctGTCTAGAGTCATGTTAGGTGG
		R: ggggaccactttgtacaagaaagctgggtTGACGTGGTGATTCTCAGTGG
*EMB1990*	2533	F: ggggacaagtttgtacaaaaaagcaggctTGTGTCATGGATTACTAATTT
		R: ggggaccactttgtacaagaaagctgggtCGATTTCTGGATTTGAGGTTG
*EMB2001*	3822	F: ggggacaagtttgtacaaaaaagcaggctCATATATGTGTTGAAAACTCA
		R: ggggaccactttgtacaagaaagctgggtGTTTGCTTGTTATATTGTGTA
*EMB2036*	3501	F: ggggacaagtttgtacaaaaaagcaggctTCGTCGCTGGTTCTATGGTTT
		R: ggggaccactttgtacaagaaagctgggtCTCTCAAGGAAACGTGCAAGA
*EMB2107*	4995	F: ggggacaagtttgtacaaaaaagcaggctCAGAGATTACAAGATATCCTG
		R: ggggaccactttgtacaagaaagctgggtACTGACTCCAGCAAAATCGGC
*EMB2736*	5372	F: ggggacaagtttgtacaaaaaagcaggctACAGGTATGGGCATCAGGTTT
		R: ggggaccactttgtacaagaaagctgggtACGAGCTCACAATCAGAGTAC
*EMB3008*	5953	F: ggggacaagtttgtacaaaaaagcaggctCTTCTGATCGGGTGCTTGATA
		R: ggggaccactttgtacaagaaagctgggtTGACTATGACGACTGTTGCTG
*GAL4*	489	F: TCAAGTGCTCCAAGAAGAAGC
		R: TGTCCAGATCGAAATCGTCT
*CRE*	1031	F: CACCATGGCCAATTTACTGACCGTAC
		R: CTAATCGCCATCTTCCAGCAG

F: forward primer. R: reverse primer. *att*B1 and *att*B2 sites are represented in lower case.

### Heat shock sector induction

Plants carrying *HS*_*pro*_ *:CRE* and pCB1-Gateway constructs combined with *emb* mutations were sowed in Petri dishes. After growing for 6 days, plates were sealed with Parafilm and submerged in water at 37 °C during 4 hours. They were put inside the plant growth chamber again and, after 5-6 days, the different lines were observed using fluorescence microscopy, in order to detect sectors with GFP signal.
